# Factors Associated With Cannabis Use During the Reproductive Cycle: A Retrospective Cross-Sectional Study of Women in States With Recreational and Medical Cannabis Legalization

**DOI:** 10.1007/s10995-021-03197-1

**Published:** 2021-06-21

**Authors:** Danica Loralyn Taylor, Janice F. Bell, Susan L. Adams, Christiana Drake

**Affiliations:** 1 Public Health Sciences Department, University of California, Davis, USA; 2 Betty Irene Moore School of Nursing, University of California, Davis, USA; 3 Department of Statistics, University of California, Davis, USA

**Keywords:** Cannabis, Prenatal, Tobacco, Legalization, Preconception, Post-partum

## Abstract

**Introduction:**

Passage of cannabis laws may impact cannabis use and the use of other substances. The suggested association is of particular concern in pregnant women where exposure to substances can cause harm to both the pregnant woman and fetus. The present study contributes to the minimal literature on factors associated with cannabis use during the preconception, prenatal, and postpartum periods including state legalization status, concurrent use of tobacco and e-cigarettes and adequacy of prenatal care.

**Methods:**

We conducted a cross-sectional analysis using combined survey data from the 2016–2018 Pregnancy Risk Assessment Monitoring System (PRAMS) collected from 36,391 women. Logistic regression was used to estimate the impact of state-legalization, adequacy of prenatal care, and other substance use on cannabis use during the preconception, prenatal, and post-partum periods.

**Results:**

In the preconception model, residence in a recreationally legal state (OR: 2.37; 95% CI, 2.04–2.75) or medically legal state (OR:3.32; 95% CI, 2.90–3.80) compared to a non-legal state was associated with higher odds of cannabis use. In the prenatal model, residence in a recreationally legal state was associated with higher odds of cannabis use (OR: 1.51; 95% CI, 1.29–1.79) whereas there was no association with residence in a medically legal state. Tobacco use including e-cigarettes and moderate prenatal alcohol use were also significantly associated with cannabis use.

**Conclusion:**

Recreational cannabis legalization is associated with the use of cannabis prior to, during, and after pregnancy. Renewed clinical and policy efforts may be warranted to update prenatal substance use prevention programs, educational campaigns, and provider education as cannabis legalization evolves.

## Significance

Previous literature shows a link between the passage of laws legalizing the use of cannabis for medical or recreational purposes and the possible effect on use rates, especially among youth. However, research into the impact of cannabis laws among pregnant women is limited. This study finds consistent associations of legalization of recreational cannabis is associated with cannabis use throughout the reproductive cycle; medical cannabis legalization was also associated with cannabis use, but only in the preconception period.

Concurrent use of tobacco products including e-cigarettes was also associated with cannabis use in this population. These findings suggested legalization may play a role in cannabis use during pregnancy and providers may want to consider how and when the council pregnant women about substance use in states with legalization.

## Introduction

In 1996, California became the first state to legalize cannabis for medical use and 28 other states have since followed suit, with eight also allowing recreational cannabis use (Smart Approaches to Marijuana, 2019). Today, cannabis is the most used illicit drug in the past year among all adults; 10–34% report use (Cerdá et al., 2019; Marijuana trends & statistics 2020; Schuermeyer et al., 2014; Yu et al., 2020) and among pregnant women 4–9% of report use (Brown et al., 2017; Crume et al., 2018; Ko et al., 2015; Metz & Stickrath, 2015; Young-Wolff et al., 2019). Cannabis use during pregnancy may have adverse effects on perinatal and child health outcomes including low infant birth weight and child mental health concerns (Conner et al., 2016; Gunn et al., 2016; Shankaran et al., 2004). Moreover, the perception of harm of cannabis use among pregnant women may be decreasing along with legalization, given that some dispensaries “recommend” cannabis to pregnant women to alleviate pregnancy-related symptoms (Dickson et al., 2018). Additionally, studies show that women who use cannabis during pregnancy are also more likely to use it in conjunction with tobacco, the most used substance during pregnancy (Conner et al., 2016; Crume, 2019; Gunn et al., 2016; Ko et al., 2015). The concurrent use of two substances is a potentially dangerous combination since tobacco and cannabis use together is associated with an increased risk of adverse birth outcomes including stillbirth and small-for-gestational age (SGA) when compared to using one substance alone (El Marroun et al., 2016; Gunn et al., 2016).

Several studies have analyzed cannabis use among pregnant and non-pregnant women. Researchers reported that low education levels, being unmarried, and using other substances all correlate with cannabis use, and very few studies examine state legalization status as a potential factor (Brown et al., 2017; Gnofam et al., 2019; Ko et al., 2015). Studies of the general population and the limited studies including pregnant women show that cannabis legalization may affect patterns of substance use behavior and in recreationally legal states show a direct correlation between cannabis legalization and increased use of cannabis (Cerdá et al., 2019; Gnofam et al., 2019; Mark et al., 2017; Schuermeyer et al., 2014).

Studies of pregnant women specifically found, those who use cannabis tend to enter prenatal care later and to use other substances along with cannabis, including tobacco (Crume et al., 2018; Ko et al., 2015). Importantly, these studies did not account for electronic cigarette (e-cigarette) use, even though studies show known health risks and use has increased markedly in the U.S. among pregnant women (Liu et al., 2019; Wagner et al., 2017; Whittington et al., 2018). Therefore, studies are needed that account for e-cigarette use when evaluating prenatal and postnatal concurrent tobacco and cannabis use. Furthermore, given the evolving cannabis policy in the U.S. and negative health impacts of both cannabis and tobacco on fetal and women’s health, current data specific to pregnant women that also account for state legalization status are crucial for advancing prenatal care and education.

The current study was designed to address these gaps. Using the Pregnancy Risk Assessment Monitoring System (PRAMS) survey, the sample included respondents from 16 states with varying types of cannabis legalization to: (1) Examine the prevalence of cannabis use during the preconception, prenatal and postpartum periods (2) Determine prevalence of co-occurring use of tobacco (including e-cigarettes) before and during pregnancy (3) Identify factors associated with preconception, prenatal, and postpartum use of cannabis including state legalization status. We hypothesized that factors associated with cannabis use during the three time periods will be like those in the general population including a higher use among women living in states with recreational cannabis legalization. Additionally, we predicted women using cannabis would be more likely to co-use other substances, particularly, tobacco.

## Methods

### Data Source and Sample

The study is a secondary analysis of cross-sectional survey data collected in the 2016 through 2018 Pregnancy Risk Assessment Monitoring System (PRAMS) by the Centers for Disease Control and Prevention (CDC) and state health departments. Based on a stratified sampling frame which selects from recent live births with complete birth certificate data, the PRAMS survey is sent to pregnant women six weeks to three months post-partum throughout the United States as a way of monitoring perinatal health behaviors and experiences and their subsequent impact on infant health outcomes (Shulman et al., 2018). Comparison between states is possible through standardization of procedures and survey instruments. The sample for the current study included women 18 and older who provided an answer to the questions about cannabis use. Women under 18 were excluded due to the potential confounding factors associated with adolescent pregnancy and their lack of legal access to cannabis. The final sample included 36,391 women living in 16 states in the United States (AK, CO, HI, KS, KY, ME, MI, MO, MT, ND, NH, SD, VT, WA, WI, WV) who were administered questions specific to cannabis use on the PRAMS survey and who gave birth between January 1st 2016 and December 31st 2018 for a weighted sample reflecting 15,486,000 women. Given the study design and possibility that women answered the survey for multiple pregnancies, prior to analysis we searched for duplicate participant identifiers and did not find repeat ids.

Women agreed to participate in the survey with the knowledge and consent that the data may be used for scientific study (Schulman et al., 2018). The study was reviewed and deemed exempt from further review by the University Institutional Review Board based on use of deidentified data. Therefore, the study was completed in accordance with the ethical standards in the 1964 Declaration of Helsinki and its later amendments.

### Measures

#### Outcomes

Individuals were classified either as cannabis users if they answered “yes” or nonusers if they answered “no” during three time periods: before pregnancy (preconception), during the last three months of pregnancy (prenatal), and 6–12 weeks after delivery (postpartum). Three prevalence outcomes were examined. (1) Cannabis use during the last three months of pregnancy (prenatal) based on the question, “At any time during the last 3 months of your recent pregnancy, did you use marijuana or hash in any form”?; (2) Cannabis use in the 12 months before pregnancy (preconception), based on the question, “At any time during the 12 months before your recent pregnancy, did you use marijuana or hash in any form”?; and (3) cannabis use since delivery (post-partum) based on the question, “At any time during the 3 months since delivery, did you use marijuana or hash in any form”? The final question regarding use after delivery was only asked in 8 of the 16 states analyzed (AK, CO, HI, ME, MI, NH, WA, & WV).

#### Sociodemographic and Other Characteristics

Socio-demographic variables included: self-reported age in years (18–19, 20–24, 25–29 = reference 30–34, 35–39, 40+), race/ethnicity (white non-Hispanic = reference, Black non-Hispanic, Hispanic, Asian/Pacific Islander, Native American/ Native Alaskan, and Other/Mixed/Unknown, education (12 years or less = reference, 13–15 years and 16 years or more) and marital status (married or not married = reference (including divorced, single widowed). Adequacy of prenatal care was categorized with the Kotelchuck Adequacy of Prenatal Care Utilization index (Kotelchuck, 1994) as inadequate (received less than 50% of expected visits), intermediate (50–79%), adequate (80–109% = reference), adequate plus (110% or more).

Cigarette smoking and e-cigarette use were combined to create a tobacco use dummy variable (1 = use; 0 = no use) based on the following questions: (1) “In the last 3 months of your pregnancy, how many cigarettes did you smoke on an average day”? (2) “In the 3 months before you got pregnant, how many cigarettes did you smoke on an average day”? and (3) “How many cigarettes do you smoke on an average day now”? (i.e., after delivery). All participants who indicated 1 or more cigarettes were coded as cigarette smokers and participants who indicated zero as nonsmokers. E-cigarette use was measured similarly based on the questions: (1) “During the 3 months before you got pregnant, on average, how often did you use e-cigarettes or other electronic nicotine products”?; and (2) In the last three months of your pregnancy, on average, how often did you use e-cigarettes or other electronic nicotine products.

Moderate alcohol use prior to pregnancy was coded as yes when participants indicated they consumed more than seven drinks in a week (as per the U.S Department of Health and Human Services definition 2020), otherwise the variable was coded as “no”.

State cannabis legalization status was categorized as recreationally legal if recreational and medical legalization was passed prior to 2016. Alaska, Colorado, and Washington had legalized medical use prior to recreational legalization and thus were deemed recreationally legal. Hawaii, Maine, Michigan, New Hampshire, and Vermont had medically legalized marijuana prior to 2016. Status was categorized as none if the state had no policies (KS, KY, MO, MT, ND, SD, WI, WV) allowing for the use of cannabis with THC (Tetrahydrocannabinol). States that allow use of the non-psycho active Cannabidiol and not THC were classified as states with no-legalization.

### Statistical Analysis

All analyses were conducted using Stata version 14 (StataCorp LP, College Station, TX) with PRAMS weights applied to account for the complex sampling design and generate estimates generalizable to pregnant women across the United States. The PRAMS weights are determined by multiplying the sampling, nonresponse, and noncoverage of the weight yields and more detailed is available elsewhere (Shulman et al., 2018). Less than 1% of respondents were missing data for any covariate; therefore, variables with missing data were imputed with the mode. All analysis was repeated with complete cases only with no substantive differences in the findings; results presented include imputed values.

The prevalence of cannabis use was estimated in each of the three time periods (preconception, prenatal and post-partum). All socio-demographic and prenatal care characteristics were summarized and compared with chi-square tests for cannabis users and non-users during each time (preconception, prenatal or postpartum). Logistic regression was used to examine each cannabis use outcome (preconception, prenatal and postpartum) as a function of state cannabis legalization status and the socio-demographic and health covariates. As a sensitivity analysis the three models were also repeated with clustering at the state-level, with no substantive differences to the reported results.

## Results

The prevalence of cannabis use at any time (preconception, prenatal, and postpartum) was 13.6 percent. Specifically, 11.0% reported preconception, 6.6% reported prenatal use during the last three months of pregnancy, and 8.0% used cannabis during the post-partum period (Table 1). Younger age, lower educational attainment, being unmarried, and ethnicity of mixed or other race were all significantly associated with higher prevalence of any cannabis use (Table 2).

**Table 1:** Unweighted prevalence of cannabis use prenatal and partum cannabis use

(n = 36319)	n	%
None	31391	79.4
Any (before, during or after)	4928	20.6
Timing of use		
Before pregnancy	3996	11.0
During pregnancy	2393	6.6
Post-partum^a^	1570	8.0
Co-use with tobacco		
Pre-pregnancy	2069	6.9
During pregnancy	1054	3.3
Post-partum^a^	1475	4.7

^a^Postpartum limited to 8 states (AK, CO, HI, ME, MI, NH, WA, & WV)

**Table 2:** Characteristics of pregnant women

Demographic characteristics	Any cannabis use before, during or after pregnancy (n = 4928)	No cannabis use before during or after pregnancy (n = 31391)	
	% (95% CI)	% (95% CI)	p-value
Age, years			
18–19	6.5 (5.9–7.3)	3.5 (3.2–3.7)	** < 0.01**
20–24	26.9 (25.3–28.6)	17.7 (17.3–18.2)	
25–29	33.1 (31.6–34.8)	31.7 (30.7–31.9)	
30–34	22.3 (21.2–23.6)	30.6 (30.0–31.3)	
35–39	9.5 (8.7–10.4)	14.4 (13.9–14.9)	
40+	1.7 (1.4–2.1)	3.1 (2.9–3.4)	
Education			
12 years or less	55.6 (54.1–57.0)	29.6 (28.9–30.3)	** < 0.01**
13–15 years	34.1 (32.7–35.5)	31.2 (30.6–31.8)	
16 or more years	10.4 (9.5–11.3)	39.2 (38.5–39.9)	
Marital status			
Not married	68.6 (67.2–70.0)	32.0 (31.1–32.7)	** < 0.01**
Married	31.4 (30.0–32.8)	68.0 (67.3–68.7)	
Race/ethnicity			
Non-hispanic white	49.4 (48.0–51.0)	52.7 (52.0–53.5)	** < 0.01**
Non-hispanic black	18.8 (17.7–19.9)	15.1 (14.6–15.6)	

Race/ethnicity	Any cannabis use before, during or after pregnancy (n = 4928)	No cannabis use before during or after pregnancy (n = 31391)	
	% (95% CI)	% (95% CI)	p-value
Hispanic	2.7 (2.2–3.2)	6.4 (6.0–6.7)	
Asian/Pacific Islander	1.6 (1.3–2.1)	6.9 (6.6–7.3)	
Native American/Alaska native	11.4 (10.5–12.4)	4.7 (4.4–5.0)	
Other/unknown/mixed	16.1 (15.1–17.1)	14.2 (13.8–14.7)	
Other characteristics			
State legalization			** < 0.01**
Recreational legalization	17.8 (16.7–18.8)	22.2 (21.7–22.8)	
Medical legalization	30.8 (29.6–32.1)	27.7 (27.1–28.3)	
No legalization	51.4 (49.9–52.8)	50.0 (49.3–50.8)	
Kotelchuck index			** < 0.01**
Inadequate	19.0 (17.6–20.0)	9.9 (9.5–10.4)	
Intermediate	11.6 (10.7–12.6)	10.3 (9.8–10.7)	
Adequate	35.4 (34.0–36.7)	44.4 (43.6–45.1)	
Adequate plus	34.1 (32.8–35.5)	35.4 (34.6–36.1)	
Tobacco use			** < 0.01**
No tobacco use	39.1 (38.7–40.5)	80.3 (79.7–80.7)	
Tobacco use	60.9 (59.5–62.3)	19.7 (19.2–20.2)	
Moderate alcohol use ^a^			** < 0.01**
No moderate alcohol use	69.2 (67.8–71.0)	66.9 (66.2–67.5)	
Moderate alcohol use before pregnancy	6.9 (6.2–7.6)	2.3 (2.1–2.5)	
Skipped or unknown	23.9 (22.7–25.1)	31.0 (30.2–31.6)	

*AIAN* american indian, alaskan native, *PI* pacific islander, *CI* confidence interval

α = 0.05

^a^Moderate alcohol use prior to pregnancy defined as consuming > seven drinks in a week

Among cannabis users during any of the three time periods, 55.6% had educational attainment of 12 years or less compared with 29.6% among non-users (p < 0.01). Women who reported cannabis use at any time also reported higher rates of inadequate prenatal care compared to women who did not use cannabis at any point (19.0 versus 9.9%; p < 0.01). Cannabis users also had a higher prevalence of using tobacco or e-cigarettes compared to women who never used cannabis (60.9 versus 19.7%; p < 0.01). Women living in recreationally legal or non-legal states had lower use rates than women living in medically legal states; however, the association was not statistically significant.

In all three multivariable models (Table 3: preconception and prenatal; Table 4: post-partum), unmarried women had higher odds of cannabis use, while being married, reporting Hispanic ethnicity, and higher parity were associated with lower odds of use. Inadequate prenatal care (compared to adequate plus) was associated with higher odds of cannabis use in the preconception (Odds Ratio [OR] 1.26, 95% CI 1.09–1.47) and prenatal (OR 1.57, CI 1.32–1.88) models.

**Table 3:** Logistic regression of cannabis use before and during pregnancy

Characteristics	Preconception		Prenatal	
	OR (95% CI)	p	OR (95% CI)	p
Age, years				
18–19	1.30 (1.05–1.62)	0.02	1.17 (0.88–1.56)	0.27
20–24	1.08 (0.91–1.19)	0.23	1.17 (0.99–1.37)	0.09
25–29	–		–	
30–34	0.95 (0.83–1.10)	0.47	0.93 (0.78–1.01)	0.41
35–39	0.96 (0.81–1.16)	0.74	0.88 (0.73–1.03)	0.33
40+	0.87 (0.62–1.22)	0.41	1.10 (0.52–1.01)	0.62
Education				
12 years or less	–		–	
13–15 years	0.89 (0.79–1.00)	0.052	0.88 (0.77–1.02)	0.096
16 or more years	0.70 (0.59–0.82)	< 0.01	0.44 (0.34–0.55)	< 0.01
Marital status				
Married	–		–	
Not married	2.23 (1.97–2.51)	< 0.01	2.29 (1.96–2.67)	< 0.01
Race/ethnicity				
White non-hispanic	–		–	
Non-hispanic black	1.98 (1.72–2.27)	< 0.01	1.19 (0.99–1.43)	0.24
Hispanic	0.58 (0.42–0.79)	< 0.01	0.59 (0.41–0.98)	0.05
Asian	0.76 (0.55–1.01)	0.08	0.58 (0.29–0.59)	0.02
AIAN	2.72 (2.27–3.28)	< 0.01	1.24 (0.97–1.56)	0.07
Other/unknown/mixed	1.46 (1.27–1.67)	< 0.01	1.2 (1.07–1.51)	0.01
Legalization status in state of residence				
Residence in a recreational legal state	2.37 (2.04–2.75)	< 0.01	1.51 (1.29–1.79)	< 0.01
Residence in a medical legal state	3.32 (2.90–3.80)	< 0.01	0.90 (0.76–1.06)	0.21
Residence in a state with no legalization	–		–	
Tobacco use(traditional and e-cigarette)				
Tobacco use	3.22 (2.93–3.53)	< 0.01	4.50 (4.00–5.00)	< 0.01
No tobacco use	–		–	
Moderate alcohol use before pregnancy				
No moderate use	–		–	
Moderate alcohol use before pregnancy	1.91 (1.56–2.38)	< 0.01	2.30 (1.81–2.92)	< 0.01
Skipped or unknown	0.33 (0.28–0.38)	< 0.01	0.40 (0.36–0.47)	< 0.01
Kotelchuck index				
Inadequate	1.26 (1.09–1.47)	< 0.01	1.62 (1.41–1.88)	< 0.01
Intermediate	1.01 (0.85–1.20)	0.87	1.37 (1.16–1.62)	< 0.01
Adequate	–		Reference	
Adequate plus	1.00 (0.89–1.19)	0.91	1.03 (0.89–1.20)	0.67
Parity				
No previous live births	–		–	
One previous live birth	0.66 (0.59–0.74)	< 0.01	0.86 (0.79–0.99)	0.05
Two previous live births	0.60 (0.52–0.71)	< 0.01	0.82 (0.68–0.97)	0.04
Three or more previous live births	0.58 (0.49–0.70)	< 0.01	0.85 (0.68–1.05)	0.13

Moderate alcohol use prior to pregnancy defined as consuming > seven drinks in a week

*AIAN* american indian, alaskan native, *PI* pacific islander, *CI* confidence interval

α = 0.05

In the preconception model (Table 3), residence in a recreationally legal state (OR 2.37, 95% CI 2.04–2.75) or medically legal state (OR 3.32, 95% CI 2.9–3.8) compared to a non-legal state was associated with higher odds of cannabis use. Other covariates associated with higher odds of use were age 18–19 years compared to 25 to 29 years (OR 1.30, 95% CI 1.05–1.62); identifying as Black non-Hispanic (OR 1.98, CI 1.27–2.27)or Native American/Alaskan compared to white, non-Hispanic (OR 2.77, CI 2.27–3.28); using tobacco prior to pregnancy (OR 3.22, CI 2.93–3.53) and reporting moderate alcohol use prior to pregnancy (OR 1.91, CI 1.56–2.38).

In the prenatal model (Table 3), residence in a recreationally legal state was associated with higher odds of cannabis use (OR 1.51, 95% CI 1.29–1.74) whereas residence in a medically legal state was not. Tobacco use (OR: 4.50; CI: 4.00–5.00) and moderate prenatal alcohol use (OR 2.30, CI 1.81–2.92) were again associated with cannabis use.

In the post-partum model (Table 4), residence in a recreationally legal state was associated with higher odds of cannabis use (OR 1.98, 95% CI 1.01–1.81) compared to residence in a non-legal state, but residence in a medially legal state was not significantly associated. Tobacco (OR 3.3, 95% CI 2.8–4.0) and moderate alcohol use prior to pregnancy (OR 2.10, 95% CI 1.54–2.86) were also associated with higher odds of post-partum cannabis use.

**Table 4:** Logistic regression of cannabis use postpartum (n = 14981)

Characteristics	Postpartum cannabis use	
	OR(95% CI)	p
Age, years		
18–19	1.17 (0.82–1.67)	0.38
20–24	1.15 (0.91–1.37)	0.29
25–29	–	
30–34	0.86 (0.69–1.07)	0.18
35–39	0.86 (0.65–1.14)	0.31
40+	1.14 (0.60–1.90)	0.61
Education		
12 years or less	–	
13–15 years	0.76 (0.64–0.91)	< 0.01
16 or more years	0.49 (0.38–0.64)	< 0.01
Marital status		
Married	–	
Not married	1.90 (1.5–2.23)	< 0.01
Race/ethnicity		
White non-hispanic	–	
Non-hispanic black	0.93 (0.73–1.17)	0.91
Hispanic	0.59 (0.39–0.86)	0.01
Asian	0.60 (0.38–0.94)	0.03
AIAN	0.86 (0.64–1.16)	0.33
Other/unknown/mixed	0.96 (0.75–1.23)	0.75

Legalization status in state of residence		
Residence in a recreational legal state	1.98 (1.26–3.80)	0.01
Residence in a medical legal state	1.27 (0.82–1.96)	0.27
Residence in a state with no legalization	–	
Tobacco use(traditional and e-cigarette)		
Tobacco use	3.36 (2.8–4.01)	< 0.01
No tobacco use	–	
Moderate alcohol use before pregnancy		
No moderate use	–	
Moderate alcohol use before pregnancy	2.10 (1.54–2.86)	< 0.01
Skipped or unknown	0.32 (0.28–0.37)	< 0.01
Kotelchuck index		
Inadequate	1.25 (0.99–1.57)	0.06
Intermediate	1.24 (90.99–1.57)	0.76
Adequate	–	
Adequate plus	0.96 (0.75–1.23)	0.97
Parity		
No previous live births	–	
One previous live birth	0.83 (0.69–0.99)	0.05
Two previous live births	0.75 (0.59–0.96)	0.03
Three or more previous live births	0.69 (0.52–0.90)	0.01

Moderate alcohol use prior to pregnancy defined as consuming > seven drinks in a week

*AIAN* american indian, alaskan native, *PI* pacific islander, *CI* confidence interval

α = 0.05

## Discussion

In a large sample of pregnant women in 16 states, we contribute estimates of the prevalence of cannabis use in the preconception, prenatal and post-partum periods finding associations with use in recreationally legal states and some associations with residence in medically legal states. The rate of cannabis use before pregnancy reported here is consistent with nationally reported rates of 10.5% of the general population using cannabis but lower than recent rates of 34% in 2020, reported by the National Institute on Drug Abuse (Marijuana trends & statistics, 2020).

To date, few studies include both recreational and medical cannabis legalization status as factors potentially influencing perinatal cannabis use. Studies that include legalization were typically limited to only one state and only addressed recreational legalization (Gnofam et al., 2019).

Several factors could account for the state legalization status and association with use found in the study. First, as states legalize cannabis, women’s perception of harm of cannabis decreases, resulting in an increase in use rates as noted in the general population (Mauro et al., 2019). To support this point, findings from a qualitative study where in-depth interviews were conducted found that 62% of women using cannabis reported that they would increase their use during pregnancy if cannabis was legalized (Mark et al., 2017). Similarly, another study found that women who used cannabis during pregnancy did not believe the substance harmful (Chang et al., 2019). Second, the opening of dispensaries following state legalization allows for better access to and promotion of cannabis use, possibly leading to use during pregnancy. In Colorado, a study of dispensaries found 69% recommended cannabis to the researcher who was claiming to be pregnant and asking for a recommendation on the use of cannabis during pregnancy (Dickson et al., 2018). Future studies are needed to test the proposed mechanisms as drivers of use among pregnant women in recreationally legal states.

The finding in this study of a higher odds of using cannabis during the preconception period in recreationally legal states and medically legal states are of particular concern given 45% of pregnancies in the United States are unplanned (Henshaw, 2011). In the cases of unplanned pregnancy, women using cannabis could unknowingly expose the embryo to cannabis derivatives like tetrahydrocannabinol (THC) during a critical period of fetal development. However, given that the preconception data for this study were collected up to twelve months before pregnancy to the study cannot accurately measure how close to conception women were using cannabis. Residence in states with medically legal cannabis was associated with higher odds of cannabis use during the preconception period but not associated with use at any other time. The difference in odds of cannabis use between medically and recreationally legal states could be explained by several factors. Provider responses to women may vary based on legalization status and could impact a pregnant woman’s choice to discontinue use early in pregnancy. A recent study in Pennsylvania found healthcare providers were much more likely to focus on legal implications of use rather than health implications when women disclosed use in pregnancy (Holland et al., 2016). In medically legal states, cannabis use is often only allowed for a limited set of medical conditions (Blumenauer, 2013). Therefore, if providers focus on the legality of use in states with more restrictions, pregnant women might be more convinced to quit using cannabis; whereas, in recreational states no “illegal use” exists and perhaps there is less pressure from providers for women to quit cannabis use. Similarly, another study found if providers did not discuss cannabis use during a visit most pregnant women assumed this meant cannabis use during pregnancy posed no health risk (Bayrampour et al., 2019). Duration of legalization may also play a role in the differences observed between recreational and medical cannabis states. Medical cannabis legalization first took place in 1996 and in the subsequent two decades resulted in the development of cannabis prevention programs specific to pregnancy, whereas, context of more recent recreational legalization are in their infancy. Further research is warranted to examine how prevention practices differ between states with recreational and medical cannabis legalization and the resultant outcomes.

As seen in other studies, the association with inadequate prenatal care and cannabis use in this study may be a result of selection bias insofar as women who use substances may not access prenatal care due to their substance use behaviors or fear of being reported. Alternatively, women using substances during pregnancy tend to be younger and with lower education attainment and may not access prenatal care due to some other external barriers irrespective of substance use (i.e. lack of trust of the health system, lack of access to care, no transportation, hiding pregnancy) and therefore continue use because they do not receive education about cessation of substances during pregnancy (Friedman et al., 2009; Hajizadeh et al., 2016; Stone, 2015). Inadequate prenatal care is associated with cannabis use across all time periods in this study suggesting a need for public health or clinical interventions prior to pregnancy. One possibility would be to consider delivering cannabis prevention education outside prenatal care through public service announcements and warning labels on legally sold cannabis products consistent with prevention strategies used for prenatal alcohol use (Barry et al., 2009). Furthermore, since the study found that parity was a protective factor against cannabis use in all three time periods, offering prevention education for women of reproductive age at any medical appointment may be an effective strategy to reach women before future pregnancies and promote abstinence from any substance use prior to conception.

Based on the review of the literature, this study is possibly the first to include e-cigarettes in the assessment of tobacco co-use with cannabis. E-cigarettes present an emerging public health crisis and are considered especially harmful during pregnancy given the increase in nicotine exposure to the pregnant woman and fetus (Liu et al., 2019; Wagner et al., 2017; Whittington et al., 2018). The odds of tobacco use in association with cannabis use were slightly higher than in other studies looking at traditional tobacco use alone (Gunn et al., 2016; Ko et al., 2015). Possibly, as e-cigarette use increases during pregnancy, there is a concomitant increase in use of cannabis especially given new technology making it easy to “vape” nicotine and cannabis together (Whittington et al., 2018). Whittington and et al., (2018) provided evidence that e-cigarette use is on the rise in pregnancy as is concurrently used with combustible tobacco which could account for the magnitude of the association found in this study. Notably, the indicator for tobacco use in this study was one or more cigarettes and did not differentiate between intensity of smoking possibly leading to an overestimation of use in our sample resulting in the higher reported odds.

Interpretation of the study findings is subject to several limitations including the cross-sectional design which precludes causal inference. In addition, the stigma associated with substance use in pregnancy may have resulted in underreporting of use and underestimation of prevalence rates, although the PRAMS computer-assisted interviews could decrease this bias to some degree (Shulman et al., 2018). The PRAMS also relies on women to recall their substance use from the past year, during the postpartum period, potentially leading to over- or under-reporting of past year use of cannabis (Bhandari & Wagner, 2006). Limitations due to the use of secondary data include the inability to measure cannabis use throughout the pregnancy and only at designated times (e.g., last three months of pregnancy) as specified in the survey questions. Finally, due to the difficulty of analyzing policies in motion given that recreational cannabis legalization is a new policy, a possibility exists that not enough time has passed to estimate the full impact of the changing policy on use rates (Pacula & Sevigny, 2014). Also, cannabis use rates may be higher in recreational or medical states prior to the passage of cannabis laws and therefore the higher rates of use were not associated with the policy change. Future studies should take advantage of additional years of post-recreational legalization data as they become available and analyze the direct impact on policies on prenatal use.

## Data Availability

Public use data for PRAMS is avaiable for dowload on the CDC website. State identifiers and paritcipant ID’s must be requested through the PRAMS coordinator.
